# Image-guided, whole-pelvic, intensity-modulated radiotherapy for biochemical recurrence following radical prostatectomy in high-risk prostate cancer patients

**DOI:** 10.1371/journal.pone.0190479

**Published:** 2018-01-10

**Authors:** Sang Jun Byun, Young Seok Kim, Hanjong Ahn, Choung-Soo Kim

**Affiliations:** 1 Department of Radiation Oncology, Dongsan Medical Center, Keimyung University School of Medicine, Daegu, Republic of Korea; 2 Department of Radiation Oncology, Asan Medical Center, University of Ulsan College of Medicine, Seoul, Republic of Korea; 3 Department of Urology, Asan Medical Center, University of Ulsan College of Medicine, Seoul, Republic of Korea; National Health Research Institutes, TAIWAN

## Abstract

**Background:**

The optimal field size of salvage radiotherapy (SRT) for biochemical recurrence, particularly for patients with high-risk prostate cancer, remains undefined. This retrospective analysis was performed to investigate oncological outcomes as well as treatment-related toxicity following salvage intensity-modulated radiotherapy (IMRT) to the whole pelvis and to compare the results with other studies implementing a small field size of the prostate bed.

**Methods:**

The medical records of 170 patients with high-risk prostate cancer who received SRT for biochemical recurrence following prostatectomy were reviewed. Whole-pelvic IMRT was administered with a median dose of 66 Gy in 30 fractions. To improve treatment accuracy, an endorectal balloon device and daily cone-beam computed tomography were utilized. Androgen-deprivation therapy combined with SRT was administered to 97 (57.1%) patients.

**Results:**

Eventually, 68 (40.0%) patients showed biochemical progression (BCP) after SRT. With a median follow-up period of 56 months, the 5-year BCP-free survival was 38.6%. The overall and cause-specific survival rates were 90.9% and 96.7%, respectively. Regarding BCP-free survival analysis, pathological T stage, persistent prostate-specific antigen (PSA) elevation after prostatectomy, and preSRT PSA level were significant prognostic factors on univariate analysis. On multivariate analysis, pathological T stage and preSRT PSA value retained their significance. Acute and late grade-3 genitourinary toxicities were observed in one (0.6%) and five (2.9%) patients, respectively. One patient each developed acute and late grade-3 gastrointestinal toxicity.

**Conclusion:**

SRT to whole pelvis using IMRT and image guidance is as safe as SRT to the prostate bed, but its efficacy should be confirmed in ongoing randomized trials. PreSRT PSA was the only controllable prognostic factor, suggesting the benefit of early SRT.

## Introduction

The incidence of biochemical recurrence (BCR) following radical prostatectomy (RP) is reported as high as 30% [1]. Salvage radiotherapy (SRT) is the only potentially curative treatment for BCR because it eradicates microscopic tumors within the radiation field, which is generally the prostate bed [2, 3]. Defining the appropriate clinical target volume (CTV) with whole-pelvic radiotherapy (WPRT) or prostate-bed radiotherapy (PBRT) remains controversial, particularly in high-risk patients. The results of the Radiation Therapy Oncology Group (RTOG) 0534 trial [4] is awaited to determine the role of elective pelvic irradiation for patients with increasing prostate-specific antigen (PSA) levels following surgery. The aim of the ongoing randomized Radiotherapy and Androgen Deprivation In Combination After Local Surgery (RADICALS) trial [5] is to evaluate the efficacy of pelvic nodal irradiation compared with PBRT.

Apart from its efficacy, a major concern of WPRT is treatment-related toxicity. With the advent of intensity-modulated radiotherapy (IMRT) and image-guided radiotherapy (IGRT), several studies have reported advantages of these methods in terms of dosimetric parameters of organs at risk, translating to a clinically significant reduction in toxicity [6, 7]. Endorectal balloon (ERB) is often used to protect the rectal wall and to achieve better reproducibility, thereby facilitating smaller margins [8, 9].

In this study, the authors reviewed the medical records of high-risk prostate cancer patients who received SRT to the elective pelvic nodal region with IMRT and IGRT, assuming that WPRT would be beneficial to high-risk patients and could be delivered safely. The primary objectives of this study were to determine biochemical progression-free survival (BCPFS) and SRT-related toxicity rates, and identify prognostic factors for BCPFS.

## Methods and materials

From 2007 to 2014, 190 consecutive patients with histologically confirmed prostate adenocarcinoma received SRT to the whole pelvis at Asan Medical Center. Of these 190 patients, 20 were excluded from this study for the following reasons: incomplete SRT (n = 3), loss to follow-up (n = 3), intermediate-risk group (n = 13), and use of three-dimensional (3D) conformal radiotherapy (RT) (n = 1). Finally, 170 patients with high-risk prostate cancer, as defined by the National Comprehensive Cancer Network guidelines [10], who received SRT with IMRT constituted the study population (S1 Table). The study protocol was approved by the local institutional review board of Asan Medical Center, and the requirement for informed consent was waived.

All patients underwent preSRT evaluation with computed tomography (CT), magnetic resonance imaging, and laboratory tests, which included PSA levels. Planning CT scan with a 2.5-mm slice thickness was performed with the patient in the supine position with the ankles immobilized. Patients were instructed to have empty bladders and rectums before CT simulation and subsequent treatment. ERB has been used since 2009 to avoid damage to the anterior rectal wall and to decrease the irradiated rectal volume according to previously reported methods [8, 11]. CTV included the prostate beds, external iliac, internal iliac, and obturator lymph nodal areas. In case of seminal vesicle invasion (pT3b), the seminal vesicle bed was included in CTV. If not involved, the seminal vesicle bed was excluded from CTV. The CTV is independent on either preSRT PSA level or ADT use. The planning target volume was contoured with a 3–7-mm extensional margin from CTV. Doses and fractionation schemes were changed during the treatment period as follows: until 2010, whole-pelvic and prostate-bed doses were 46 and 66 Gy, respectively, in 2 Gy per session followed by a boost of 4 Gy for gross local recurrence if any. Since 2011, whole pelvic and the prostate-bed doses were 44 and 66 Gy, respectively, in 2.2 Gy per fraction and followed by a boost of 6.6 Gy for gross lesions. RT plans were approved when at least 95% of the planning target volume received the prescribed dose and maximum dose heterogeneity was <7%. Dose constraints for organs at risk were as follows: (1) for the rectum, <20% of the volume should receive >60 Gy and <50% of the volume should receive >50 Gy; (2) for the bladder, <40% of the volume should receive >60 Gy and <60% of the volume should receive >50 Gy; and (3) for the small bowel, <150 cc should receive >45 Gy. Cone-beam CT was utilized weekly (until 2008) or daily (from 2009) to verify the ERB location and enhance setup reproducibility.

The patients were examined by radiation oncologists once every week during the course of SRT and then evaluated with a PSA test by radiation oncologists and urologist at 3-month intervals after completion of SRT for 2 years, and at least every 6 months thereafter. Acute complications were defined as events occurring during the course of SRT and within 3 months after completion of SRT, whereas late complications were defined as those occurring 3 months after SRT. The National Cancer Institute’s Common Terminology Criteria for Adverse Events version 4.02 and the RTOG morbidity grading scale for urinary frequency, nocturia, dysuria, and urgency were used to grade toxicities.

Two distinct definitions were used in the present study to avoid confusion, BCR and biochemical progression (BCP), where BCR refers to PSA elevation >0.2 ng/mL with a consecutive increase following RP, whereas BCP refers to PSA levels >0.2 ng/mL and successive elevation following SRT in high risk prostate cancer patients. Patients who received androgen-deprivation therapy (ADT) with no evidence of BCP after SRT were not counted as BCP events, but censored at the time of ADT initiation. Biochemical progression-free survival (BCPFS) was calculated from SRT initiation to the BCP confirmation after SRT or date of censor. Overall survival (OS) was calculated from SRT initiation to the date of death or last follow-up and cause-specific survival (CSS) was calculated from SRT initiation to the date of prostate cancer-related death or last follow-up. The Kaplan–Meier method and log-rank test were used to construct and compare survival curves, respectively. A Cox proportional hazards model was used for multivariate analysis. The chi-square test was used to evaluate differences among factors between two groups. A p-value of <0.05 was considered to indicate statistical significance and all statistical analyses were performed using IBM^®^ SPSS^®^ for Windows, version 22.0 (IBM corporation, Armonk, NY).

## Results

The patient characteristics are listed in Table 1. The median patient age was 66 years. The median initial (before RP) and preSRT PSA levels (regardless of neoadjuvant ADT) were 18.53 and 0.65 ng/mL, respectively. All of the patients had at least one high-risk attribute. The Gleason score was 8–10 in 111 (65.3%) patients and pathological T stage was ≥pT3a in 145 (85.3%). Nineteen (12.6%) patients had pathologically proven metastatic lymph node(s). The median number of harvested lymph nodes was five with an interquartile range of three to eight. Neoadjuvant, concurrent, or adjuvant ADT was administered with SRT in 97 (57.1%) patients.

**Table 1:** Patient characteristics.

Variables		Number of patients (%)
Age (years)	Median (range)	66 (47–82)
Initial PSA level (ng/mL)	Median (IQR)	18.53 (9.9–37.2)
PSA level before SRT (ng/mL)	Median (IQR)	0.65 (0.46–1.00)
Gleason score	Median (range)	8 (7–10)
	7	59 (34.7)
	8–10	111 (65.3)
pT stage	pT1c—2c	25 (14.7)
	pT3a	74 (43.5)
	pT3b—4	71 (41.8)
pN stage	Negative	151 (88.8)
	Positive	19 (11.2)
Number of harvested lymph nodes	Median (IQR)	5 (3–8)
Extracapsular extension	Absent	25 (14.7)
	Present	145 (85.3)
Seminal vesicle invasion	Absent	99 (58.2)
	Present	71 (41.8)
Surgical margin	Negative	56 (32.9)
	Positive	114 (67.1)
Lymphovascular invasion	Absent	103 (60.6)
	Present	67 (39.4)
Roach score for LNI	Median (IQR)	35.1 (24.8–45.5)

Abbreviations: PSA = prostate-specific antigen; IQR = interquartile range; SRT = salvage radiotherapy; LNI = lymph node involvement.

**Fig 1:**
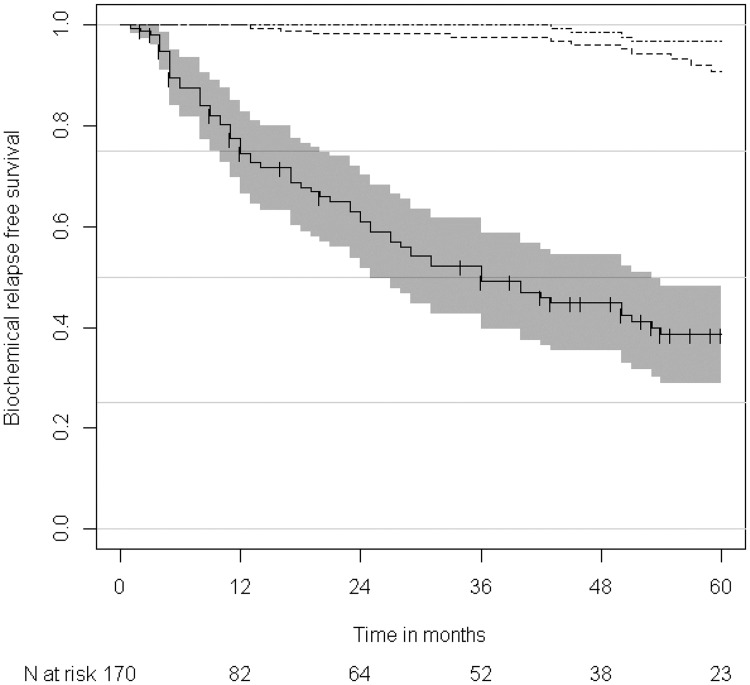
Kaplan–Meier estimates of biochemical progression-free survival (BCPFS, Solid line), overall survial (OS, Long dashed line), and cause-specific survival (CSS, Dash-dotted line). Number at risk for biochemical progression-free survival is indicated.

**Fig 2:**
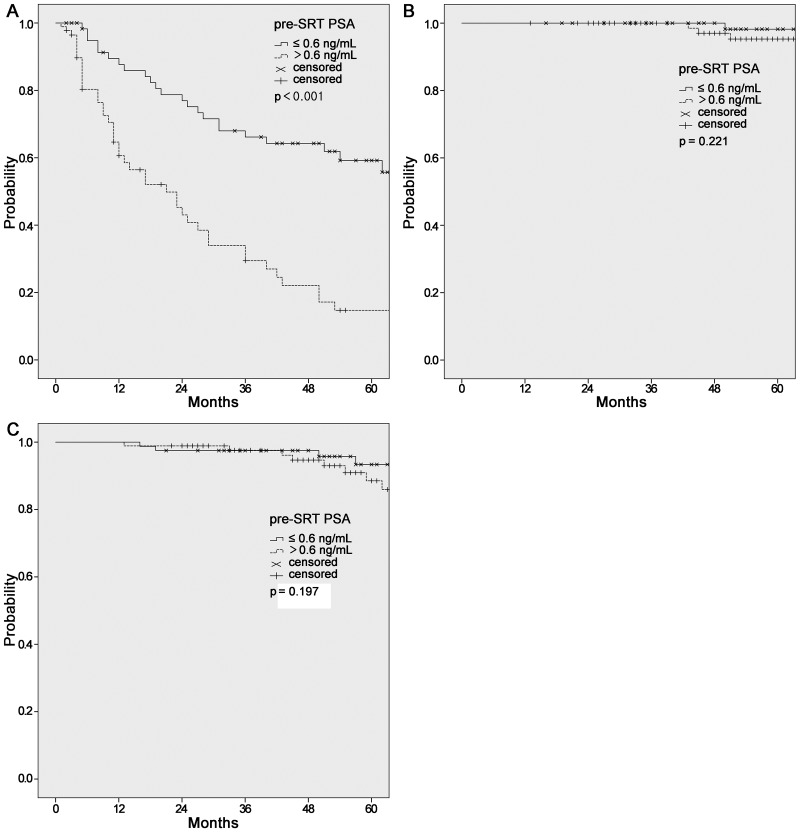
Kaplan–Meier estimates of biochemical progression-free survival (BCPFS, A), cause-specific survival (CSS, B), and overall survial (OS, C) according to preSRT PSA level.

The median follow-up time was 56 months (range, 13–106) and BCP was confirmed in 68 (40.0%) patients during follow-up. The 2-year and 5-year BCPFS rates after SRT were 61.0% and 38.6%, respectively. The 5-year OS and CSS rates were 90.9% and 96.7%, respectively (Fig 1). In total, 16 deaths were observed, including eight from other comorbidities, such as idiopathic pulmonary fibrosis or other malignancies. On univariate analysis for prognostic factors for BCPFS, preSRT PSA level, pathological T stage, lymphovascular invasion (LVI), and persistently elevated PSA after RP were statistically significant. The details are depicted in Fig 2 and Table 2. On multivariate analysis, pathological T stage (95% CI = 0.34–0.96; HR = 0.57, *p* = 0.033) and PSA level before SRT (95% CI = 0.20–0.55; HR = 0.33, *p* < 0.001) were statistically significant prognostic factors for BCPFS.

**Table 2:** Univariate analysis of prognostic factors of BCPFS, OS, and CSS.

Variables	No. (%)	5Y BCPFS	*p*-value	5Y OS	*p*-value	5Y CSS	*p*-value
PSA before SRT (ng/mL)							
≤ 0.6	80 (47.1)	59.2%	<0.001	93.4%	NS	98.2%	NS
>0.6	90 (52.9)	14.7%		88.5%		95.3%	
Gleason score							
7	59 (34.7)	46.5%	NS	100%	0.033	100%	NS
8–10	111 (65.3)	33.7%		85.7%		94.8%	
pT stage							
T1c–3a	99 (58.2)	45.8%	0.006	93.2%	NS	98.6%	0.089
T3b–4	71 (41.8)	28.7%		87.4%		93.9%	
pN stage							
Negative	151 (88.8)	40.3%	0.084	90.1%	NS	96.4%	NS
Positive	19 (11.2)	14.3%		100%		100%	
LVI							
Negative	103 (60.6)	45.4%	0.039	94.5%	0.006	97.4%	NS
Positive	67 (39.4)	27.4%		85.0%		95.5%	
PSA elevation after RP							
Delayed	90 (52.9)	57.0%	<0.001	92.8%	NS	98.4%	0.093
Persistent	80 (47.1)	19.5%		88.6%		95.0%	
ADT							
Yes	97 (57.1)	24.0%	NS	90.8%	NS	96.4%	NS
No	73 (42.9)	47.1%		91.6%		96.9%	

Abbreviations: BCPFS = biochemical progression-free survival; OS = overall survival; CSS = cause-specific survival; PSA = prostate-specific antigen; SRT = salvage radiotherapy; NS = not significant; LVI = lymphovascular invasion; RP = radical prostatectomy; ADT = androgen deprivation therapy.

Treatment-associated toxicity profiles are summarized in Table 3. Acute genitourinary (GU) toxicity of any grade was observed in 43 (25.3%) patients. The most common acute GU complications were nocturia and urgency. Acute gastrointestinal (GI) toxicity of any grades was reported in 39 (22.9%) patients. Mild bloating and gas distension frequently occurred, but usually subsided within 1 month after completion of SRT with or without conservative treatment. Late GU toxicity was observed in 43 (25.3%) patients, whereas grade-3 complications occurred in only five (2.9%). Hematuria was the most common grade-3 late GU toxicity. Most late GI toxicities were diarrhea and proctitis. Only one patient experienced severe rectal bleeding as a late toxicity.

**Table 3:** Acute and late toxicities.

Toxicity	Grade 1	Grade 2	Grade 3	Total
Acute GU	31 (18.2%)	11 (6.5%)	1 (0.6%)	43 (25.3%)
Acute GI	26 (15.3%)	12 (7.1%)	1 (0.6%)	39 (22.9%)
Late GU	22 (12.9%)	16 (9.4%)	5 (2.9%)	43 (25.3%)
Late GI	6 (3.5%)	1 (0.6%)	1 (0.6%)	8 (4.7%)

Abbreviations: GU = genitourinary; GI = gastrointestinal.

## Discussion

Although the prostate bed has been a standard field of SRT, WPRT is often used, particularly for high-risk patients, to irradiate pelvic lymph nodes [12–14], which may harbor occult metastasis, as demonstrated by a recent study using nanoparticle-enhanced magnetic resonance imaging [15]. As depicted in Table 1, the median probability of pelvic lymph node involvement according to the Roach formula [16] was 35.1%. Moreover, the median number of harvested lymph nodes was only five in this study, which is lower than in previous surgical series [17, 18]. The lower number of dissected lymph nodes could lead to the underestimation of lymph-node involvement risk. Thus, high-risk prostate cancer patients routinely receive WPRT in our institution.

The 5-year BCPFS, the primary endpoint in the present study, was 38.6%, which seems to be inferior to that reported in previous studies [13, 14, 19]. Spiotto et al. [13] compared the results between WPRT and PBRT in an adjuvant or salvage setting, and reported a 5-year BCPFS rate after WPRT of 47% in 72 patients. However, these 72 patients included high-risk and intermediate-risk patients who received adjuvant RT or SRT. They argued in favor of WPRT for high risk patients, only with concurrent use of ADT, as in the RTOG 94–13 trial. King et al. [14] compared WPRT and PBRT and demonstrated a significant difference in favor of WPRT (5-year BCPFS of 54% vs. 37%, *p* = 0.023, on univariate analysis). In that study, the Gleason score, initial PSA, and T stage were more favorable attributes than in the present study. Another study of hypofractionated IMRT in an adjuvant or salvage setting to the prostate bed also showed a 4-year BCPFS rate of 75% [19]. That study also included patients with more favorable attributes in terms of Gleason score and T stage than in the present study. Apart from the different inclusion criteria of each study, another hurdle to compare the results was the variable definitions of BCP after SRT. The definition of BCP in the present study was a PSA elevation >0.2 ng/mL and successive elevation following SRT. This definition is in accordance with one study [20], whereas other studies used a different definition, a detectable PSA confirmed on repeat testing or rising on subsequent testing [13, 14]. Moghanaki et al. reported an excellent 5-year BCPFS rate of >60% in high-risk patients; however, the adopted definition was milder (post-SRT nadir + 0.2 ng/mL and a subsequent rise) [20]. Taking into account patients with higher-risk attributes who received SRT and the stricter BCP definition of the present study, the authors believe that the BCPFS rate in the current study is at least comparable to others. Moreover, the median PSA before SRT in the present study was 0.65 ng/mL which was higher compared to those of the “very early” SRT studies. Recently, there are a few papers advocating early or very early SRT [21, 22]: in one study, it was reported that preSRT PSA less than 0.2 ng/mL was associated with reduction in distant metastasis by half compared to the traditional definition of early SRT of 0.2 to 0.5 ng/mL [21]. In addition, using a large database from 2,460 patients, Tendulkar, et al. demonstrated that preSRT PSA was a significant prognostic factor for biochemical control as well as distant metastasis, emphasizing very early initiation of SRT even less than 0.2 ng/mL [22].

**Fig 3:**
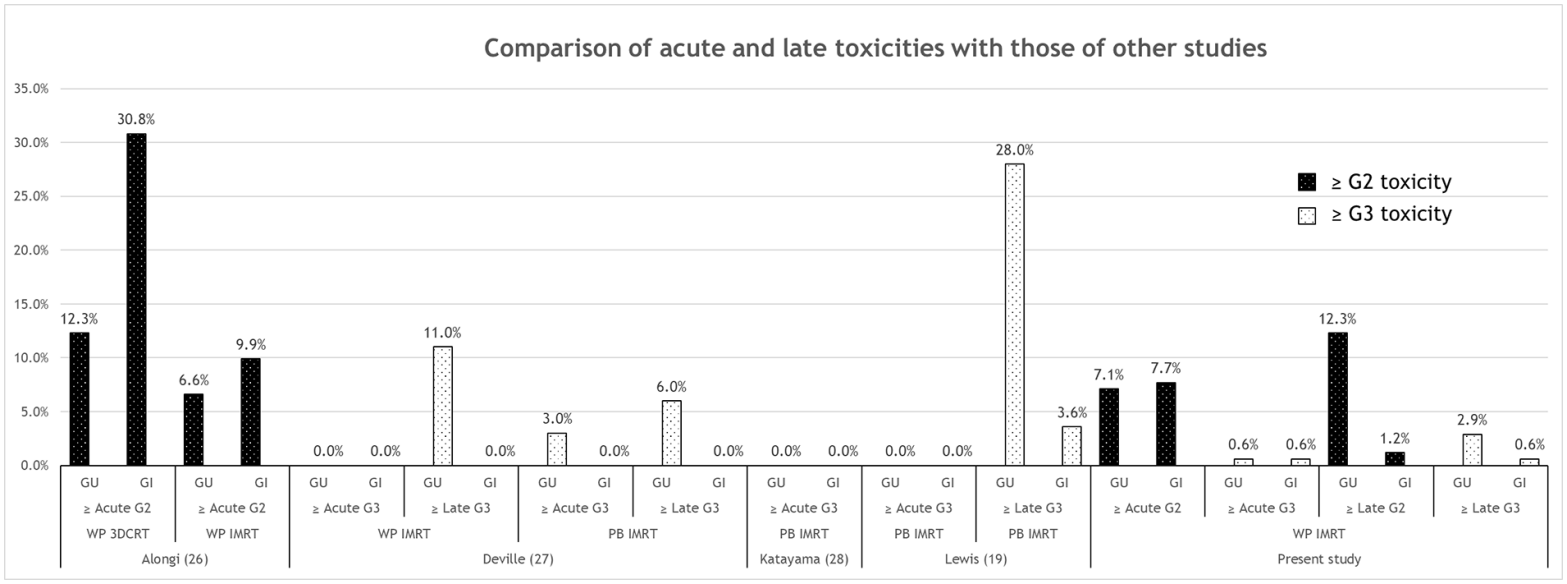
Comparison of acute and late toxicities with those of other studies. Abbreviations: RT = radiotherapy; GU = genitourinary; GI = gastrointestinal; WP = whole pelvis; 3DCRT = three-dimensional conformal radiotherapy; G = grade; IMRT = intensity-modulated radiotherapy; PB = prostate bed.

With regard to treatment-related toxicity, PBRT was reported to be well-tolerated with low rates of severe GU and GI complications even with a 3D conformal technique (3DCRT) [23, 24]. However, increased toxicity with WPRT, as demonstrated in the RTOG 94–13 trial [25], was the major concern of WPRT. With the advent of IMRT, several studies have compared the toxicity of whole-pelvic IMRT and whole-pelvic 3DCRT [26] or whole-pelvic IMRT to prostate-bed IMRT [27] in an adjuvant or salvage setting (Fig 3). Alongi et al. [26] reported that the incidence of ≥grade-2 acute GU and toxicity of the upper and lower GI tracts were lower in patients treated with IMRT than 3DCRT, and the most significant reduction occurred in upper GI toxicity. In several recent trials using moderate hypofractionation with a daily fraction size of 2.5 and 3 Gy in postprostatectomy IMRT [19, 28], CTV encompassed the prostate bed only and did not include the pelvic nodal area. There was no instance of ≥grade-3 acute GU or GI toxicity, but ≥grade-3 late GU toxicities were reported in 28% patients in the former study. Because of the large pelvic lymphatic area covered by CTV, mild hypofractionated IMRT of 2.2 Gy per day was adopted in the current study. The incidence of toxicity seems to be similar or lower than in those other studies, which can be attributed to the use of ERB in 93.5% patients and meticulous IGRT [8]. The only patient with severe rectal bleeding after SRT in the present study underwent SRT without the use of ERB in 2008, as it was not introduced yet. It could be assumed that this complication resulted from not using ERB. Several studies also reported a decrease in toxicity with the use of IGRT [29, 30].

ADT was administered concurrently with SRT in 97 (57.1%) patients in the current study. There was no significant difference in BCPFS (24.0% vs. 47.1%) or OS (90.8% vs. 91.6%) and CSS (96.4% vs. 96.9%) by the use of ADT. These results are contradictory to the findings of Spiotto et al. [13], who claimed that a benefit WPRT over PBRT was observed only with the use of ADT. Because only a limited number of studies has investigated salvage WPRT with or without ADT, the role of concomitant ADT remains elusive until the results of ongoing randomized trials are published. Regarding the synergistic effect between RT and ADT, it is well known that ADT inhibits non-homologous end joining, an important DNA repair process [31]. Additionally, based on the recently published article, it was also demonstrated that RT resulted in androgen receptor (AR) upregulation in various prostate cancer models *in vitro* and *in vivo* [32], providing another evidence of synergism between ADT and RT. In that paper, they measured AR-regulated hK2 protein and proved that AR upregulation occurred in about 20% of patients receiving definitive RT.

The present study has several limitations, including its retrospective design, which may inherently have introduced potential biases, and the short follow-up period, which may have been insufficient to assess long-term toxicity. Also, there was no control group who received PBRT in the present study because the authors thought WPRT would be necessary in this population. Despite these drawbacks, this study has several unique attributes; 1) the patients were homogeneous, i.e., only high-risk patients were included, 2) as a single institutional study, a consistent SRT technique and follow-up policy were implemented, 3) whole-pelvic SRT was administered safely with the use of IMRT, IGRT, and ERB, and 4) mild hypofractionation was utilized considering the low alpha/beta ratio and resource efficiency. In addition, to the best of our knowledge, this is the largest analysis of SRT to the pelvis in high-risk prostate cancer patients.

## Conclusions

SRT to the whole pelvis using IMRT and meticulous image guidance is as safe as SRT to the prostate bed, but its efficacy should be verified by ongoing randomized trials. PreSRT PSA was the only controllable prognostic factor, suggesting the benefit of early SRT.

## Supporting information

S1 Table Demographic and clinicopathologic data. Data extracted medical records from 170 patients with high risk prostate cancer who underwent salvage radiotherapy for biochemical recurrence following radical prostatectomy.
(XLSX)
